# Ptprd deficiency promotes tau hyperphosphorylation and impairs cognitive function in aged mice

**DOI:** 10.1186/s40659-025-00607-4

**Published:** 2025-05-06

**Authors:** Analía Foncea, Nayhara Franchini, Isidora Tobar, Sebastián Thienel, Ignacio N. Retamal, Gonzalo I. Cancino, Francisca Cornejo

**Affiliations:** 1 Centro de Biología Integrativa, Facultad de Ciencias, Universidad Mayor, Santiago, Chile; 2 Centro de Oncología de Precisión, Escuela de Medicina, Facultad de Medicina y Ciencias de la Salud, Universidad Mayor, Santiago, Chile; 3 Laboratorio de Neurobiología, Facultad de Ciencias Biológicas, Pontificia Universidad Católica de Chile, Santiago, Chile

**Keywords:** PTPRD, Tau phosphorylation, ABL1, Tauopathy, Cognitive impairment, Microgliosis, Synaptic dysfunction

## Abstract

**Background:**

Tau phosphorylation is a tightly regulated process that ensures proper neuronal function. Indeed, hyperphosphorylation of tau closely contributes to neuronal dysfunction leading to neurodegenerative diseases, including tauopathies, which are characterized by excessive and aberrant tau phosphorylation and cognitive decline. Therefore, it is important to understand how to regulate its phosphorylation. In this regard, the protein tyrosine phosphatase receptor delta (PTPRD) has been genetically implicated in tau pathology in humans, but the mechanisms underlying its role in tau regulation remain unclear. This study investigates the impact of Ptprd deficiency on tau phosphorylation, cognitive function, neuroinflammation, and synaptic markers in aging mice.

**Results:**

Mice lacking Ptprd showed increased tau phosphorylation at multiple sites associated with its pathological aggregation. This effect was accompanied by the activation of the tau-related kinase Abl1, particularly in the hippocampus. Behavioral assessments revealed significant impairments in learning and memory, demonstrating the functional impact of these alterations. Moreover, Ptprd knockout mice showed increased microgliosis in both the entorhinal cortex and the hippocampus, suggesting a pro-inflammatory response. Furthermore, the synaptic protein PSD95 was also reduced in the cortex, indicating potential synaptic dysfunction.

**Conclusions:**

The loss of Ptprd leads to increased tau phosphorylation, cognitive impairments, microgliosis, and synaptic alterations in older mice. Our findings also suggest that Ptprd plays a critical role in maintaining tau homeostasis through the Abl1 kinase. This indicates a new potential therapeutic approach for tauopathies, where PTPRD could serve a protective role against tau-related pathologies and may act as a key modulator in disease progression.

## Background

Tauopathies are a group of neurodegenerative disorders characterized by diverse clinical presentations, ranging from cognitive and behavioral impairments to movement disorders [1–3], including Alzheimer’s disease (AD), frontotemporal dementia, progressive supranuclear palsy, corticobasal degeneration, and chronic traumatic encephalopathy, among others [3–5]. The main pathological hallmark of these disorders is the abnormal accumulation of tau protein in neurons and glial cells [6]. This accumulation leads to the formation of neurofibrillary tangles (NFTs) and other tau inclusions consisting of aggregated hyperphosphorylated tau protein [6–9] which can lead to synaptic impairment and ultimately, neuronal cell death [1, 3, 10]. Tau is a protein mainly found in neurites and axons of neurons, where it favors tubulin polymerization and stabilizes neuronal cytoarchitecture [8], participating in neuronal morphology maintenance, neurite and axon growth, and axon transport, among others, having an important role in neuronal function and cell viability [9]. Tau microtubule-affinity is regulated post-translationally by phosphorylation, where tau is typically found in a constant dynamic equilibrium between a microtubule-associated and a cytoplasmic-soluble form depending on its phosphorylation status [6]. In pathological conditions, such as in tauopathies, impairments in the tau phosphorylation/dephosphorylation balance can induce its abnormal phosphorylation, inducing microtubule dissociation, intracellular aggregation, and pathological NFT formation [11]. NFTs impair synapses and axonal transport, induce oxidative stress, and promote neuronal loss as well [6, 12].

Among the pathological mechanisms that disrupt the balance in tau phosphorylation and dephosphorylation, leading to the formation of NFTs, is the dysregulation of kinases and phosphatases associated with tau [11], such as glycogen synthase kinase-3β (Gsk3β), cyclin-dependent kinase 5 (Cdk5), Mitogen-Activated Protein Kinase 15 (Mapk15), ABL proto-oncogene 1 (Abl1), and protein phosphatase 2 A (PP2A) [13–16]. Also, recent studies have found a novel phosphatase that could be contributing to tauopathy development, the Protein Tyrosine Phosphatase Receptor Delta (PTPRD) [17–21].

PTPRD, also known as PTPδ or Ptp-delta, is a transmembrane protein that belongs to the family of type IIa tyrosine phosphatases, which in the central nervous system is expressed in neurons and glial cells participating in neurogenesis, axonal growth, and synaptogenesis [22]. Alterations in the expression of Ptprd have been associated with an aberrant brain cortical development [23–25] and with several brain pathologies such as autism spectrum disorder, schizophrenia, and obsessive-compulsive disorder, among others [26–34], suggesting that loss of PTPRD is associated with brain impairments. Interestingly, increasing evidence has shown a direct link between PTPRD and tauopathy. It has been reported that patients with sporadic AD have *PTPRD* deletions [17]. Additionally, genome-wide association studies involving older individuals without dementia at the time of enrollment—who later developed various neuropathological changes associated with aging, such as NFTs, amyloid-beta (Aβ) plaques, and Lewy bodies—revealed that the presence of postmortem NFTs was significantly related to single nucleotide polymorphisms (SNPs) in the *PTPRD* gene locus [18, 19]. Furthermore, an RNA-seq study demonstrated a significant correlation between *PTPRD* variants and the burden of hyperphosphorylated tau and cognitive impairments. This suggests that impaired expression of *PTPRD* in the human brain may increase susceptibility to NFT formation, independently of amyloid burden, in cases of sporadic AD [20]. These findings align with studies in models of tau pathology using *Drosophila melanogaster*, where the expression of *Lar*, the ortholog of *PTPRD*, was significantly decreased with aging and directly associated with tau-induced toxicity [21, 35]. Although PTPRD downregulation has been associated with tau phosphorylation and NFT formation, the role of PTPRD in the development and progression of tauopathies has not been characterized.

Therefore, we investigated whether the absence of Ptprd disrupts tau phosphorylation status in the mouse cortex and hippocampus, which are critical regions in the development and progression of tauopathies [36, 37]. Using Ptprd knockout (KO) mice, we show that the absence of Ptprd leads to increased tau phosphorylation in the brain cortex and hippocampus at 6 and 18 months. Additionally, it results in an increased activation of Abl1 tau kinase, impaired learning and memory, increased microgliosis, inflammation, and decreased levels of the synaptic marker PSD95. Consequently, our study supports the idea that reduced expression of *PTPRD* is a risk factor for the development of tauopathies, as human genetic studies suggest, and states the groundwork for future experiments to examine the detailed molecular mechanisms that underlie this phenomenon.

## Methods

### Animals

The C57BL/6N-A < tm1Brd > Ptprd < tm2a(KOMP) Wtsi>/WtsiOrl mice strain was purchased from Wellcome Trust Sanger Institute and maintained as heterozygous. The cross between heterozygous mice allowed the Ptprd^+/+^ (Ptprd WT), Ptprd^+/−^ (Ptprd HET), and Ptprd^−/−^ (Ptprd KO) mice to be obtained. The following primers were used to identify the three genotypes mentioned above: Ptprd_111547_F: 5’-TCACCTCGCTGTTCTTCCTG-3’; Ptprd_111547_R: 5’-CTTCTCAGTGCCCAACCCTC-3’; CAS_R1_Term: 5’-TCGTGGTATCGTTATGCGCC-3’. Mice were housed in groups of up to 6 animals with free access to rodent chow and water in a 12-h dark-light cycle room at 22 °C. All animal procedures were approved by the Animal Ethics Committee of Universidad Mayor, and both female and male mice were used in this study.

For ex-vivo analysis, mice were sacrificed at 6 or 18 months old and transcardially perfused with phosphate-buffered saline (PBS). The brains were extracted and split in half, and each hemibrain was used for immunofluorescence or molecular biology experiments.

### Western blot

Tissue samples from the hippocampus and brain cortex were microdissected on ice, and tissue identity was confirmed by gross morphological/anatomical landmarks. Samples were lysed in ice-cold radioimmunoprecipitation assay (RIPA) buffer (Thermo). Protein was quantified by the BCA Kit (Pierce), and 50 µg of protein lysates were run on 10% sodium dodecyl sulfate-polyacrylamide gel electrophoresis (SDS-PAGE) gels. Western blot analyses were performed as described [38]. The following antibodies were used: AT8 (recognizing Ser-202- and Thr205-phosphorylated tau, 1:500; Thermo); AT100 (recognizing Ser-214- and Thr-212-phosphorylated tau, 1:500; Thermo); AT180 (recognizing Thr-231- and Ser-235-phosphorylated tau, 1:500; Thermo); anti-pS396 (recognizing Ser-396-phosphorylated tau, 1:1000; Merck); anti-pS404 (recognizing Ser-404-phosphorylated tau, 1:1000; Merck); anti-pS422 (recognizing Ser-422-phosphorylated tau, 1:1000; Invitrogen); Tau5 antibody that recognizes total tau protein (1:1000; Invitrogen); anti-pAbl1 (recognizing Tyr412-phosphotylated Abl1, 1:1000; Sigma); anti-pGsk3β (recognizing Ser9-phosphotylated Gsk3β, 1:1000; Cell Signaling Technology); anti-pCdk5 (recognizing Tyr15-phosphotylated Cdk5, 1:250; Santa Cruz Biotechnology); anti-Abl1 (K12, 1:1000; Santa Cruz Biotechnology); anti-Gsk3β (H-76, 1:1000; Santa Cruz Biotechnology); anti-Cdk5 (C-8, 1:500; Santa Cruz Biotechnology); anti-PSD95 (1:1000; Neuromab); and anti-HSP90 (1:1000; Santa Cruz Biotechnology). Secondary horseradish peroxidase (HRP)-conjugated antibodies (1:5000) were obtained from Thermo. ImageJ (NIH, Bethesda, MA, USA) software was used for densitometry quantitative analysis.

### Morris water maze test

The Morris water maze (MWM) test was performed to evaluate spatial learning and memory as previously described [25]. During the learning phase, the animals were trained to locate a 9 cm diameter platform positioned 1 cm below the water’s surface. This training occurred over 4 consecutive days, with 4 trials each day, in a 1.2 m diameter circular pool filled with white-painted water maintained at 23 ± 2 °C. Each trial concluded when the animals found the platform and stayed there for 10 s. The learning phase measured the time it took for the animals to reach the platform, which was determined by averaging the latency times from the 4 daily trials for each mouse. On the fifth day, in the memory phase, the test was performed by removing the platform and allowing free swimming for 60 s. The time spent in the zone where the platform was allocated, the distance traveled within the platform zone, and the swimming speed were measured. The behavior was monitored during all tasks using an automatic tracking system (ANY-maze video tracking software, Stoelting Co, Wood Dale, IL, USA).

### Immunofluorescence

Mice hemibrains were fixed in 4% paraformaldehyde overnight at 4 °C. The next day, the brains were dehydrated in 20%, 25%, and 30% sucrose solutions at 4 °C for 24 h each. After dehydration, brains were embedded in the optimal cutting temperature compound (OCT; Sakura) and stored at -80 °C. Next, 18 μm coronal slices were obtained in a cryostat, mounted in positively charged microscope slides, and stored at -80 °C until use. For immunofluorescence, brain sections were washed with 1X TBS and permeabilized with TBS − 0.3% Triton X-100 for 30 min. The tissues were then incubated in TBS with 5% BSA and 0.3% Triton X-100 for 1 h as a blocking solution. Brain slices were incubated overnight with primary antibodies in a blocking solution at 4 °C. Then, the sections were washed with TBS and incubated with secondary antibodies in a blocking solution at room temperature for 1 h. After TBS washes, sections were counterstained with Hoechst 33,258 and mounted in Dako Mounting Medium (Agilent).

To evaluate microglia number and soma size, rabbit anti-Iba1 antibody (1:500, Fujifilm Wako Chemical) was used. The secondary antibody was Alexa Fluor 488-conjugated donkey anti-rabbit (1:1000, Thermo). Digital image acquisition was performed using LAS X software (Leica Microsystems) on a Leica DMi8 microscope. Photomicrographs were obtained from the entorhinal cortex and hippocampus, and cell number and soma area quantification were performed using the ImageJ software (NIH, Bethesda, MD, USA) “Analyze particles” tool, with a particle size threshold of 20 µm^2^ to exclude unspecific background noise.

### RT-qPCR

RNA from cortical and hippocampal tissues was extracted with E.Z.N.A Total RNA I kit (Omega Bio-Tek), and cDNA was synthesized from 1 µg of RNA using the iScript Reverse Transcriptase Kit (Bio-Rad) according to the manufacturer’s protocols. Quantitative PCR was performed using SsoAdvanced universal SYBR green supermix (Bio-Rad). *Rplp0* ribosomal protein expression was used as an endogenous control for the 2^−ΔΔCt^ relative expression analysis. All reactions were performed in triplicate. Quantitative PCR was performed and analyzed using the CFX96 Touch Real-Time PCR Detection System (Bio-Rad). The primers used were the following: IL1β forward: 5’-GCAACTGTTCCTGAACTCAACT-3’, IL1β reverse: 5’-ATCTTTTGGGGTCCGTCAACT-3’, RPLP0 forward: 5’-CTGCTGAACATGCTGAACATC-3’, and RPLP0 reverse: 5’-GTCGAGCACTTCAGGGTTAT-3’.

### Statistical analysis

All data were collected from at least four independent experiments. In all cases, results are presented in the graphs as the mean ± SEM. Normality of the data was assessed using the Shapiro-Wilk test. Comparisons between two groups were performed using the Mann-Whitney test for non-parametric data or the unpaired t-test for parametric data. Multiple group comparisons were analyzed using two-way ANOVA, followed by Tukey’s multiple comparisons test. All statistical analyses were conducted with GraphPad Prism version 6.0.0 for Windows (GraphPad Software). A p-value < 0.05 was considered statistically significant.

## Results

### Ptprd absence leads to aberrant Tau phosphorylation and Abl1 kinase activation in the aged brain

**Fig. 1:**
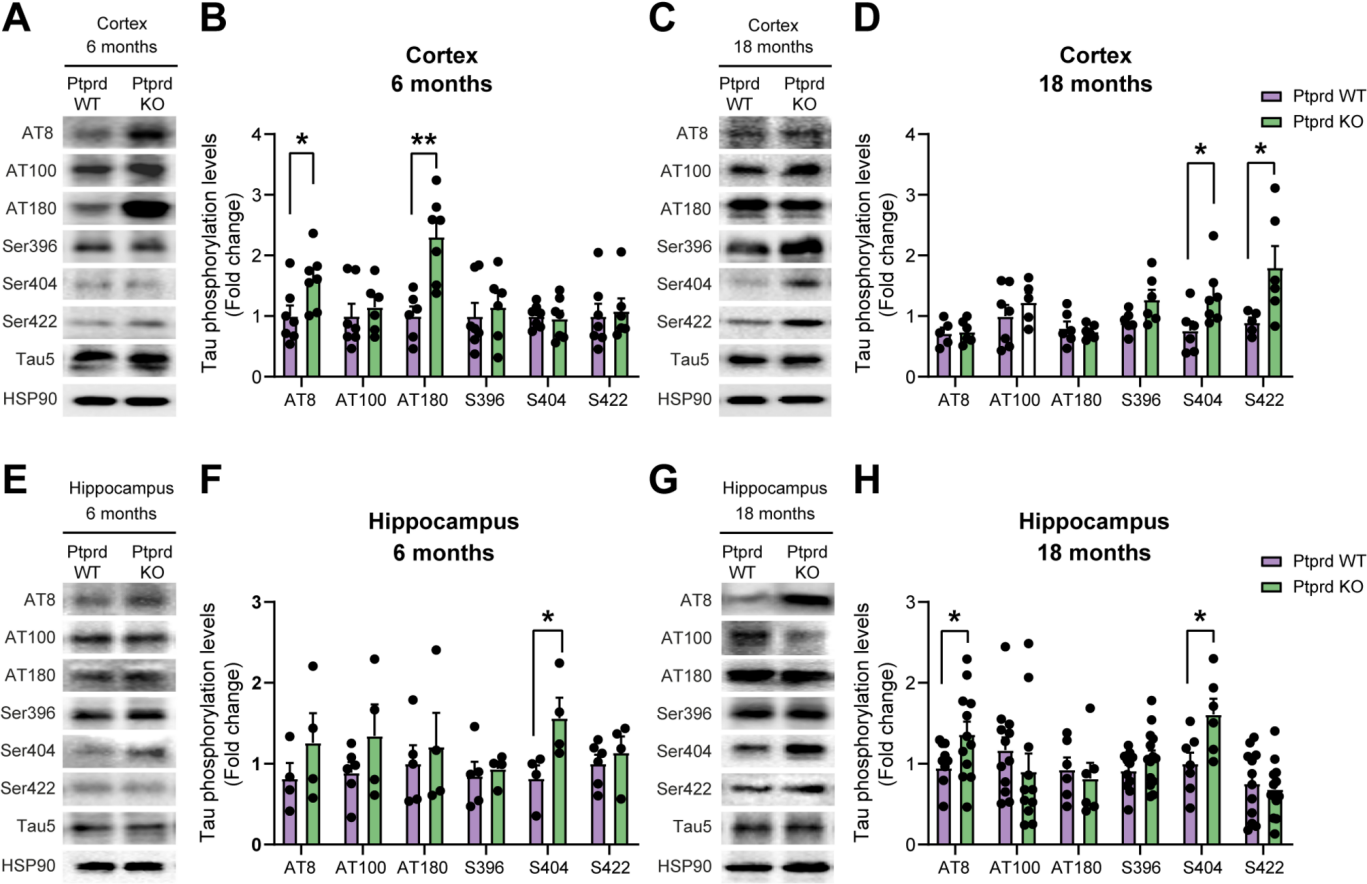
Ptprd absence causes tau hyperphosphorylation in the mouse brain. Western blot analysis of cortical (**A**, **C**) and hippocampal lysates (**E**, **G**) from 6-month and 18-month-old Ptprd WT and Ptprd KO mice, probed with AT8, AT100, AT180, pSer396, pSer403, or pSer422 antibodies to detect phosphorylated tau, and with an antibody against total tau (Tau5) and HSP90 as a loading control. Quantification by scanning densitometry of the relative level of tau phosphorylation/total tau in Western blot analysis was performed in cortical lysates at 6 months (**B**) and 18-months-old mice (**D**) and in hippocampal lysates at 6 months (**F**) and 18-months-old (**H**). *p < 0.05; *n* = 4–12

It was previously reported that in humans, variants in *PTPRD* are consistently associated with increased tau phosphorylation and cognitive impairments [17–21]. To determine whether the absence of Ptprd has an impact on tau phosphorylation status, we performed western blot analysis using the 6 phospho-tau antibodies AT8, AT100, AT180, pSer396, pSer404, and pSer442, all of which recognize different phosphorylated tau epitopes, on cortical and hippocampal lysates from 6-month- and 18-month-old Ptprd WT and Ptprd KO mice (Fig. 1). As a control, we used Tau5 antibody to probe total tau protein expression and HSP90 as a loading control. Since we did not find obvious differences in tau phosphorylation levels between females and males (data not shown), mice from both sexes were included in all experiments.

Our results showed an increase in tau phosphorylation detected by AT8 and AT180 antibodies in the brain cortex of 6-month-old Ptprd KO mice (Fig. 1A-B) and by pSer404 and pSer442 antibodies in 18-month-old Ptprd KO mice (Fig. 1C-D). Moreover, densitometry analysis of phosphorylated tau also demonstrated a significant increase in phosphorylation of the Ser404 residue in the hippocampus of 6-month-old animals (Fig. 1E-F). Furthermore, tau phosphorylation, detected by AT8 and pSer404 antibodies, was also increased in the hippocampus of aged Ptprd KO mice compared to Ptprd WT mice (Fig. 1G-H). This data shows that the lack of Ptprd results in abnormal tau phosphorylation at various residues.

**Fig. 2:**
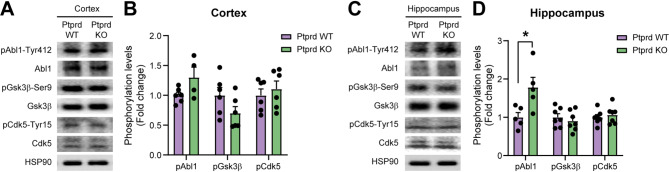
Ptprd loss causes Abl1 kinase activation in the mouse hippocampus. Western blot analysis of cortical (**A**) and hippocampal lysates (**C**) from 18-month-old Ptprd WT and Ptprd KO mice, probed with antibodies for Abl1 phosphorylated at tyrosine 412, Gsk3β phosphorylated at serine 9, or Cdk5 phosphorylated at tyrosine 15. To assess the relative levels of kinase activation, the same lysates were analyzed on a western blot using antibodies that recognized total Abl1, Gsk3β, or Cdk5. The lysates were also analyzed for levels of HSP90 as a loading control. Quantification of the relative levels of Abl1, Gsk3β, and Cdk5 phosphorylation was assessed by densitometry of cortical (**B**) and hippocampal western blots (**D**). *p < 0.05; *n* = 4–7

Then, as tau has not been described as a substrate of Ptprd, we investigated the activation status of known tau kinases in Ptprd KO mice. To assess this, we analyzed the activation of Abl1, Gsk3β, and Cdk5, which are associated with tau phosphorylation and NFT formation in tauopathies [6]. We performed western blot analyses from cortical and hippocampal lysates of 18-month-old Ptprd WT and Ptprd KO mice using the antibodies anti-phospho-Abl1 (Tyr412), anti-phospho-Gsk3β (Ser9), and anti-phospho-Cdk5 (Tyr15) (Fig. 2). Note that for Gsk3β, Ser9 phosphorylation is decreased when the kinase is activated, while for Abl1 and Cdk5, tyrosine phosphorylation is increased when the kinases are activated. Densitometry analysis normalized to total protein shows a mild increase in Abl1 kinase activation in the Ptprd KO cortex (Fig. 2A-B), while it is significantly activated in the Ptprd KO hippocampus compared to Ptprd WT mice (Fig. 2C-D). Thus, Ptprd absence increases tau phosphorylation and Abl1 activation.

### Ptprd KO mice exhibit impairments in learning and memory

**Fig. 3:**
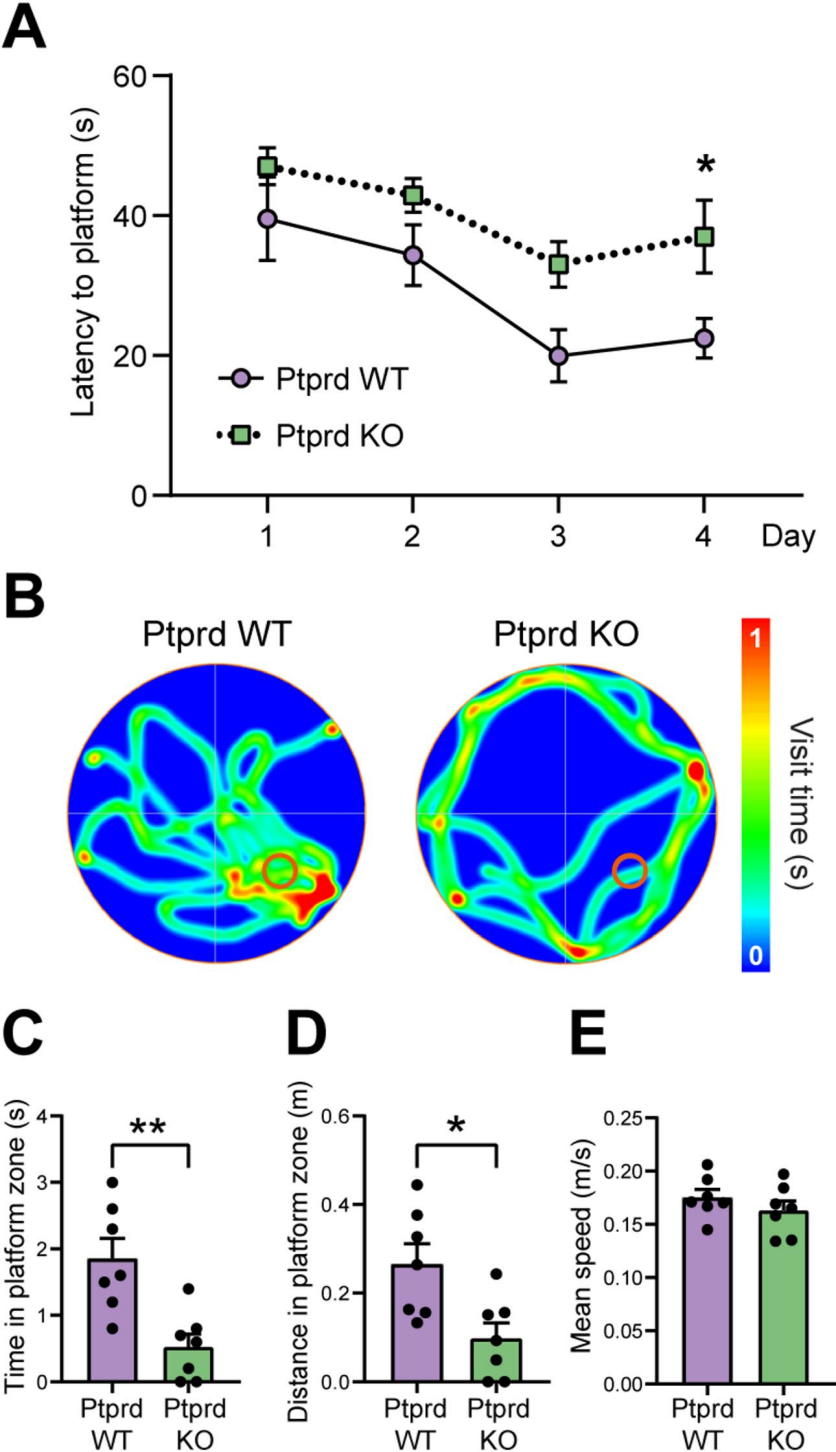
Ptprd KO mice show impairments in learning and memory. 18-month-old Ptprd WT and Ptprd KO mice were tested in the MWM test, and the latency time to find the platform during the 4 days of training was assessed (**A**). On day 5, memory was evaluated by analyzing the trajectory of mice in the platform zone. A representative heat map of Ptprd WT and Ptprd KO mice trajectory is shown in (**B**, platform represented in the right bottom), which was analyzed to quantify the time in the platform zone (**C**), the distance traveled inside the platform zone (**D**), and the mean swimming speed (**E**). *p < 0.05, **p < 0.01; *n* = 7

As *PTPRD* variants have been previously associated with cognitive impairments such as observed in tauopathies [17–20], we evaluated whether Ptprd KO mice showed impairments in learning and memory with the MWM test in 18-month-old mice (Fig. 3). We found that Ptprd KO exhibited an increased latency escape compared to Ptprd WT mice on the fourth day of training (Fig. 3A). Then, on the fifth day the platform was removed, and memory was evaluated. We found that 18-month-old Ptprd KO mice have memory impairment (Fig. 3B) as they spent less time (Fig. 3C) and traveled less distance (Fig. 3D) in the area where the platform was located compared to Ptprd WT mice. To discard that these behavioral impairments could be due to motor deficits, we evaluated the mean speed of swimming, finding no significant differences between genotypes (Fig. 3E).

### Ptprd KO mice show microgliosis, inflammation, and reduced PSD95 levels

**Fig. 4:**
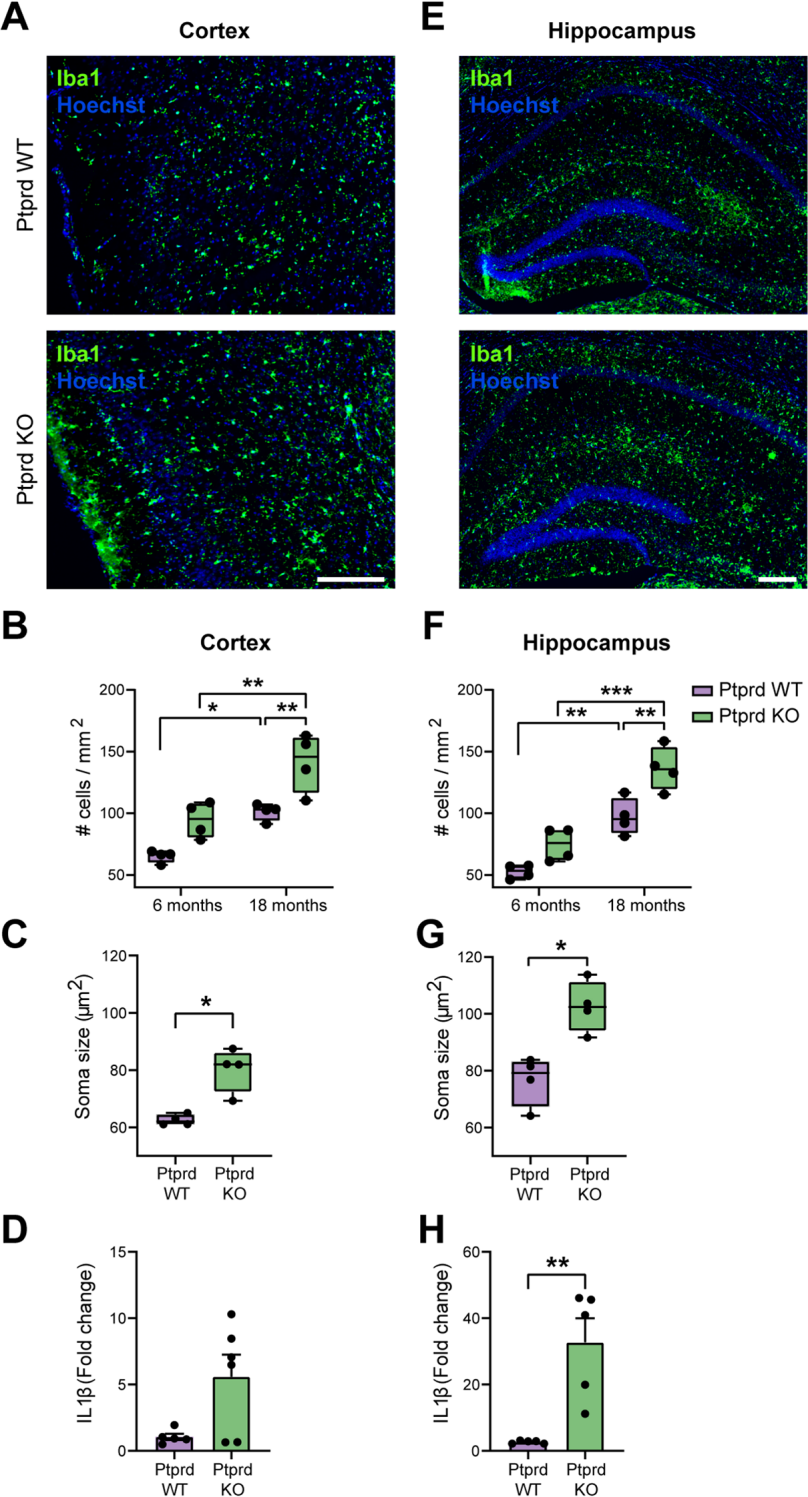
Ptprd KO mice show increased neuroinflammation in the entorhinal cortex and hippocampus. Iba1-positive cells (green) were immunolabeled in the brain entorhinal cortex (**A**) and hippocampus (**E**) of Ptprd WT and Ptprd KO mice at 18 months of age, and sections were counterstained with Hoechst 33,258 (blue). The total number of microglia per mm^2^ was quantified in 6- and 18-month-old brain entorhinal cortex (**B**) and hippocampus (**F**). The mean microglial soma size was measured in the cortex (**C**) and hippocampus (**G**) at 18 months. IL1β mRNA expression was measured by qPCR in cortical (**D**) and hippocampal samples (**H**) from 18-month-old mice. Scale bar = 200 μm. *p < 0.05, **p < 0.01, ***p < 0.001; *n* = 4-6

To characterize some of the cellular and molecular alterations induced by the loss of Ptprd and the increased tau phosphorylation, we first analyzed the inflammatory status of Ptprd KO brains by assessing microglia and IL1β expression. Since tau hyperphosphorylation induces microgliosis, which is often observed as increased cell proliferation [39, 40], we quantified the number of microglia in the dorsolateral entorhinal cortex of 18-month-old Ptprd WT and Ptprd KO mice (Fig. 4A-B). Although the number of microglia significantly increased in older Ptprd WT and Ptprd KO animals, Ptprd KO animals show a substantial increase in the number of microglia in the cortex at 18 months of age compared to WT animals (Fig. 4A-B). Since the inflammatory phenotype of microglial cells often correlates with the size of their cell bodies, where pro-inflammatory microglia tend to have an amoeboid morphology, and therefore larger cell bodies [41], we analyzed the area of ​​microglial somas in the cortex of 18-month-old animals, age at which we observed significant changes between genotypes. Our results showed that Ptprd KO microglia have larger soma areas compared to Ptprd WT microglia in the cortex at 18 months (Fig. 4C).

To evaluate the inflammatory state of the studied tissues, we analyzed the expression of IL1β by qPCR. We found that Ptprd KO mice tend to show an increase in the expression of this cytokine in the cortex at 18 months (Fig. 4D).

To confirm this result, we also performed a similar analysis in the hippocampus. We found an increase in the number (Fig. 4E-F) and soma size (Fig. 4G) of microglia in the hippocampus of 18-month-old Ptprd KO mice compared to Ptprd WT mice. Also, we found a significant increase in the expression of the proinflammatory cytokine IL1b (Fig. 4H), suggesting a proinflammatory environment in the hippocampus of aged Ptprd KO mice.

**Fig. 5:**
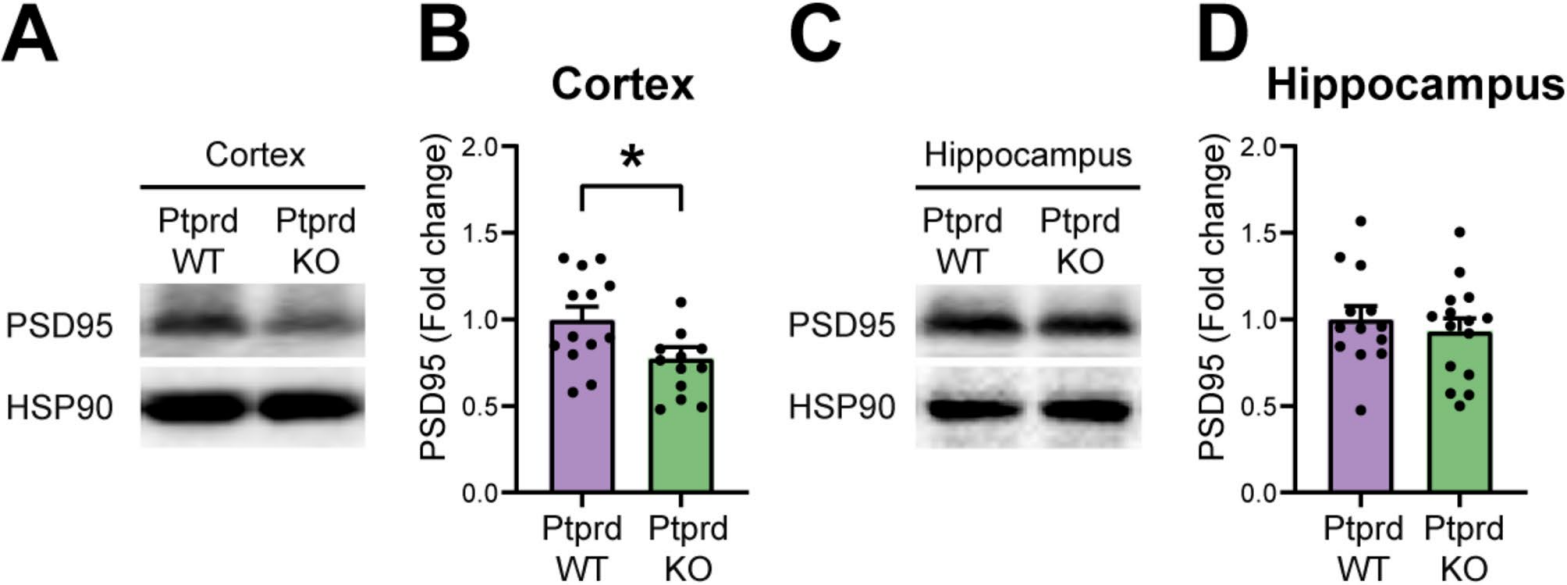
Loss of Ptprd reduces PSD95 levels in the brain cortex of older mice. Western blot analysis of cortical (**A**) and hippocampal lysates (**C**) from 18-month-old Ptprd WT and Ptprd KO mice probed for PSD95. The same lysates were also analyzed for HSP90 as loading control. Quantification of the relative levels of PSD95 was assessed by densitometry of cortical (**B**) and hippocampal western blots (**D**).*p < 0.05; *n* = 12–15

Finally, we evaluated whether PSD95 protein levels were disrupted in the cortex and hippocampus of aged Ptprd KO mice, as an indirect measurement of synaptic deficits [42, 43]. We found reduced protein levels of PSD95 in the brain cortex of Ptprd KO mice (Fig. 5A-B), with no differences in the hippocampus compared with Ptprd WT at 18 months old (Fig. 5C-D). Therefore, cognitive impairments observed in Ptprd KO mice are accompanied by increased microgliosis and IL1β expression, and reduced PSD95 levels in the aged brain.

## Discussion

There is growing evidence that links PTPRD to tauopathy in humans [17–20]. In this study, we showed that the absence of Ptprd leads to increased tau phosphorylation, activation of Abl1 tau kinase, cognitive impairments, microgliosis, neuroinflammation, and reduced PSD95 levels in aged mice. These findings provide further evidence of the role of PTPRD in tau pathology and suggest that its loss may be a contributing factor in the development of tauopathies.

Our results indicate that the loss of Ptprd promotes tau hyperphosphorylation at multiple sites that have been implicated in tau misfolding and the formation of NFTs in AD and other tauopathies [7, 11, 44]. Interestingly, the increase in tau phosphorylation at Ser404 was already evident at 6 months in the hippocampus of Ptprd KO mice, suggesting that Ptprd loss may predispose neurons to early tau pathology. Also, we found an increased tau phosphorylation in the brain cortex in 6-month-old Ptprd KO mice detected by AT8 and AT180 antibodies. However, this increase was lost by 18 months, when we only observed a significant increase in pSer404 and pSer422. Ser404 and Ser422 phosphorylation are often linked to more advanced tau pathology, whereas AT8 (Ser202/Thr205) and AT180 (Thr231) phosphorylation are typically considered markers of early tau hyperphosphorylation and dysfunction [45]. Thus, this pattern suggests a shift from soluble hyperphosphorylated tau (detected at 6 months) to more aggregated, late-stage tau (at 18 months) in Ptprd KO mice. If tau inclusions are forming, AT8 and AT180 may no longer be efficiently detected, while Ser404 and Ser422 persist as markers of pathological tau accumulation. Although it is unclear whether Ptprd regulates NFT formation, performing Sarkosyl fractionation [46] or using the P301L mice, an animal model that generates NFTs [47], would elucidate tau differential solubility and aggregation with age in Ptprd KO mice.

Since tau has not been described as a substrate for Ptprd, we evaluated the activation of some tau kinases. Specifically, we assessed Abl1, Gsk3β, and Cdk5 phosphorylation levels with phospho-antibodies associated with their activity. Our results showed a significant increase in Abl1 phosphorylation (Tyr412) in Ptprd KO mice hippocampus, accompanied by hyperphosphorylation of tau at Ser/Thr residues associated with tauopathies (AT8, pSer404). ABL1 is a non-receptor tyrosine kinase known to regulate tau phosphorylation and contributes to tau-mediated neurotoxicity [38, 48]. Although we do not provide direct biochemical evidence of Abl1 interaction or dephosphorylation by Ptprd in this study, there is evidence suggesting that Ptprd can regulate Abl1 phosphorylation [49]. Furthermore, it would be interesting to evaluate tau phosphorylation at Tyr18 or Tyr394, residues that Abl1 can directly modify and that could function as a priming event for subsequent Ser/Thr phosphorylation, contributing to tau pathology. Moreover, the increase in tau phosphorylation observed in the cortex was not associated with Abl1 activation in the cortex. This effect could be methodological since the cortical samples used for the western blot analysis were from lysates of the entire cortex. Therefore, by using lysates from specific cortical regions affected by tauopathies, such as the entorhinal cortex, we might have seen more significant differences between the Ptprd WT and Ptprd KO animals. On the other hand, the hippocampus is one of the earliest and most affected regions in tauopathies [36, 37], suggesting that it could be more susceptible to disruptions in kinase/phosphatase regulation, making it more sensitive to the absence of Ptprd.

Although we identified changes in Abl1 and tau phosphorylation, we cannot definitively establish that tau hyperphosphorylation is due solely to Abl1 dysregulation. Since we did not observe changes in the classic markers of Gsk3β (pSer9) and Cdk5 (pTyr15) activation, the question arises about how Ser/Thr phosphorylation is elevated. One possibility is that the initial phosphorylation of Tau at Tyr (pTyr18, pTyr394, etc.) changes tau’s conformation, facilitating the action of Ser/Thr kinases without necessarily requiring increases in their overall activation. Likewise, Gsk3β and Cdk5 are proline-directed kinases widely associated with pathological tau phosphorylation. Gsk3β can be regulated by phosphorylation at both Ser9 (whose phosphorylation decreases its activity) and Tyr216 (whose phosphorylation can enhance it), whereas Cdk5 requires binding to its cofactors, p35 or p25, for its activation [50]. Our analyses did not find significant changes in the “canonical” activation markers (pSer9-GSK3β and pTyr15-Cdk5), suggesting that the observed tau hyperphosphorylation could be due to alternative regulations. Such regulations could include phosphorylation of Gsk3β at Tyr216, changes in the subcellular localization of the kinases, or modifications in the accessibility of tau to these complexes in specific neuron compartments. A further possibility is that Cdk5 activity is altered not at the level of Tyr15 phosphorylation but through changes in its subcellular distribution or cofactor availability. Cdk5 is activated by binding to p35 (or its calpain-cleaved product p25), and its compartmentalization to dendrites or the nucleus can markedly influence tau phosphorylation patterns [51, 52]. Thus, even with an unchanged pTyr15 signal in whole-tissue lysates, local enrichment of Cdk5–p35/p25 complexes near microtubules could selectively enhance Ser202/Thr205 (AT8) and Thr231 (AT180) phosphorylation as we observed at 6 months old Ptprd KO mice. Future fractionation or high-resolution imaging studies—together with p35/p25 quantification—will be required to determine whether PTPRD loss triggers a redistribution of Cdk5 that contributes to the transient AT8/AT180 elevation we observe. Alternatively, other tyrosine kinases (e.g., Fyn) or other post-translational events could be involved. These findings, therefore, must be understood in the context of a largely correlational study, and functional and subcellular localization assays are necessary to further understand the mechanistic role of Ptprd.

In addition to Abl1, there is evidence that Ptprd can dephosphorylate tyrosine kinases, such as TrkB or even Fyn, another important regulator of tau phosphorylation and synaptic dynamics [24, 53]. Moreover, hyperactivated Abl1 or Fyn could propagate to downstream Ser/Thr pathways— for example, by increasing calpain-dependent p25 generation that boosts Cdk5 activity, or by favoring Gsk3β activation through Tyr216 phosphorylation— thereby providing an additional route to the heightened AT8/AT180 signals detected at six months [54–56]. We do not rule out that the phenotypes observed in Ptprd KO mice result from combined alterations in multiple signaling pathways. Future studies could address Fyn and tau phosphorylation at other relevant residues to clarify the role of other tyrosine kinase-mediated pathways.

Cognitive impairments observed in the MWM test also suggest a role of Ptprd in maintaining neuronal function. The increased latency to escape and reduced time and distance traveled in the platform area indicate deficits in spatial learning and memory, characteristic of tau-mediated neurodegeneration [37]. Notably, all mice displayed comparable swimming speed during MWM, suggesting that motor deficits were not a major confounding factor. These findings are aligned with previous reports linking *PTPRD* genetic variants to cognitive decline in humans [18, 20]. However, we cannot conclude that this cognitive dysfunction is exclusively due to tau hyperphosphorylation. Indeed, the reduction in PSD95 protein levels observed in the cortex of Ptprd KO mice suggests synaptic alterations that could underlie the observed cognitive impairments. PSD95 is a critical scaffolding protein involved in synaptic stability and plasticity, and its loss has been associated with cognitive decline in AD and other neurodegenerative disorders [42, 43]. Our results are consistent with previous studies showing a positive correlation between tau pathology and synaptic loss [57]. Although PSD95 levels may decrease due to neurodegeneration, our analyses revealed no changes in the number of neurons in the brain regions studied in Ptprd KO mice (data not shown). The selective reduction in cortical PSD95, rather than in the hippocampus, may indicate region-specific synaptic vulnerability, where cortical circuits could be more susceptible to Ptprd-dependent scaffolding or synaptic regulation. Besides, Fyn-mediated phosphorylation of tau is known to mislocalize tau to dendritic spines and destabilize PSD95-containing complexes [58, 59], offering a mechanistic explanation for the selective cortical loss of PSD95 that we observe. This difference could also be related to neurodevelopmental impairments induced by Ptprd absence (and independently of tau pathology), since we have previously shown that Ptprd KO mice have an impaired cortical development that persist until adulthood [23–25], which could be altering synaptogenesis and/or synaptic stability.

Additionally, our data showed increased microgliosis in the entorhinal cortex and hippocampus of Ptprd KO mice, which was assessed as an increased microglial number and soma area. We also observed increased IL1β mRNA expression in both tissues, while a significant increase was only shown in the hippocampus, suggesting that neuroinflammation might contribute to the observed cognitive deficits. Microgliosis is a well-known hallmark of tauopathies and has been implicated in exacerbating tau pathology through inflammatory signaling cascades [60, 61]. While our data suggests an overall pro-inflammatory state, we did not assess canonical microglial activation markers (e.g., iNOS, Cox-2) [62], and future work will elucidate whether this microglial phenotype is predominantly neurotoxic or neuroprotective. The link between Ptprd loss and microgliosis remains to be fully elucidated. Still, tau hyperphosphorylation induced by Ptprd deficiency could lead to a pro-inflammatory environment that drives microglial activation. However, microglial activation induced by an impaired interaction with neurons caused by the absence of Ptprd cannot be ruled out and needs further study.

## Conclusions

Taken together, our results suggest that the absence of Ptprd leads to tau hyperphosphorylation at sites critical for pathological aggregation, accompanied by Abl1 activation, neuroinflammation, and synaptic alterations in the aging brain. These changes are associated with cognitive impairments, suggesting that Ptprd loss could significantly contribute to the progression of tauopathies by promoting abnormal tau phosphorylation. Furthermore, the increased expression of proinflammatory cytokines such as IL1β and the decreased PSD95 in the cortex point to a compromised inflammatory and synaptic state that could exacerbate neuronal decline.

However, our data do not exclude the involvement of other Ptprd pathways or substrates. Indeed, a complete elucidation of the molecular mechanism will require further studies, including co-immunoprecipitation assays between Ptprd and Abl1, direct measurements of tau phosphorylation on tyrosine residues (e.g., Tyr18 or Tyr394), and the use of specific Abl1 inhibitors in Ptprd-null mice. Such approaches will be essential to confirm whether a Ptprd–Abl1–tau axis exists that directly explains tau hyperphosphorylation and the resulting cognitive dysfunction.

In summary, our findings consolidate the evidence that Ptprd plays a regulatory role in tau homeostasis and propose new lines of research focused on developing potential therapeutic strategies to control pathological tau phosphorylation in neurodegenerative diseases.

## Data Availability

The datasets used and/or analyzed during the current study are available from the corresponding author upon request.

## Abbreviations

Aβ: Amyloid-β Peptide
AD: Alzheimer’s Disease
ABL1: Abelson Tyrosine-Protein Kinase 1
CDK5: Cyclin-Dependent Kinase 5
GSK3β: Glycogen Synthase Kinase-3β
MWM: Morris Water Maze
NFTs: Neurofibrillary Tangles
PSD95: Postsynaptic Density Protein 95
PTPRD: Protein Tyrosine Phosphatase Receptor Delta
